# Population-level effects of fitness costs associated with repressible female-lethal transgene insertions in two pest insects

**DOI:** 10.1111/eva.12159

**Published:** 2014-05-01

**Authors:** Tim Harvey-Samuel, Thomas Ant, Hongfei Gong, Neil I Morrison, Luke Alphey

**Affiliations:** 1 Department of Zoology, University of OxfordOxford, UK; 2 Oxitec Ltd, Milton ParkOxford, UK

**Keywords:** fitness costs, genetic engineering, insect, integrated pest management, release of insects carrying a dominant lethal, transgenic

## Abstract

Genetic control strategies offer great potential for the sustainable and effective control of insect pests. These strategies involve the field release of transgenic insects with the aim of introducing engineered alleles into wild populations, either permanently or transiently. Their efficacy can therefore be reduced if transgene-associated fitness costs reduce the relative performance of released insects. We describe a method of measuring the fitness costs associated with transgenes by analyzing their evolutionary trajectories when placed in competition with wild-type alleles in replicated cage populations. Using this method, we estimated lifetime fitness costs associated with two repressible female-lethal transgenes in the diamondback moth and olive fly as being acceptable for field suppression programs. Furthermore, using these estimates of genotype-level fitness costs, we were able to project longer-term evolutionary trajectories for the transgenes investigated. Results from these projections demonstrate that although transgene-associated fitness costs will ultimately cause these transgenes to become extinct, even when engineered lethality is repressed, they may persist for varying periods of time before doing so. This implies that tetracycline-mediated transgene field persistence in these strains is unlikely and suggests that realistic estimates of transgene-associated fitness costs may be useful in trialing ‘uncoupled’ gene drive system components in the field.

## Introduction

Genetic engineering has enabled the development of new methods for the sustainable control of insect pests. Such genetic pest management strategies use the mating behavior of a pest species to introduce novel heritable traits into wild target populations. To date, such traits include lethal phenotypes and decreased vector competence (or refractoriness to infection) (Sinkins and Gould 2006; Alphey et al. 2013; Alphey 2014). The success of such an approach depends on the field performance of the engineered insects, especially in terms of finding and mating with wild counterparts. Estimation of fitness costs associated with transgenic strains, particularly those affecting mating ability, has therefore become an area of considerable research interest (Catteruccia et al. 2003; Irvin et al. 2004; Moreira et al. 2004; Marrelli et al. 2006, 2007; Lambrechts et al. 2008; Scolari et al. 2008; White et al. 2010; Harris et al. 2011, 2012; Massonnet-Bruneel et al. 2013; Paton et al. 2013).

One genetic pest management approach, called release of insects carrying a dominant lethal (RIDL), involves the RIDL transgene that renders progeny nonviable in the field (Thomas et al. 2000). Transgene-induced lethality is made conditional through the use of the ‘tet-off’ gene expression system (Gossen and Bujard 1992), which is repressed by provision of tetracycline (TC), or suitable analogs, to the insects – usually as a supplement in the larval diet (‘on-tet’). Unlike the classical autocidal control approach, the sterile insect technique (SIT) RIDL does not require sterilization by irradiation, which can have negative impacts on sexual competitiveness and field survival (Shelly et al. 1994; Lance et al. 2000; Alphey et al. 2010).

A female-specific variant of RIDL (fsRIDL) limits engineered lethality to females, allowing male-only production and mortality of female progeny in the field (Schliekelman and Gould 2000; Fu et al. 2010; Black et al. 2011; Ant et al. 2012; Jin et al. 2013). Male-only releases can offer improved per-male efficiency (McInnis et al. 1994; Rendón et al. 2000, 2004) and reduced female-specific damage in the field, such as oviposition damage by fruit flies or biting by mosquitoes. In addition, field survival of fsRIDL male heterozygotes may provide an insecticide resistance management strategy through introgression of susceptibility alleles into wild populations (Alphey et al. 2007, 2009).

In the fsRIDL strains investigated here – OX4319L-Pxy diamondback moth (*Plutella xylostella* L.) (Jin et al. 2013) and OX3097D-Bol olive fruit fly (*Bactrocera oleae* Gmelin) (Ant et al. 2012) – engineered lethality is limited to females through the use of sex-alternate splicing sequences from sex determination genes: in OX4319L-Pxy *doublesex* from the pink bollworm, *Pectinophora gossypiella*; in OX3097D-Bol *transformer* from Mediterranean fruit fly (Medfly, *Ceratitis capitata* Wiedemann). In OX4319-Pxy, insertion of the tTAV transactivator coding sequence from the tet-off system into the female-specific exon of a *dsx* minigene results in tTAV transcription in females only. In OX3097D-Bol, the tTAV insertion in an exon of a *tra* minigene is in frame with female transcripts and out of frame with those of males. As engineered TC-repressible lethality in these strains is dependent on the expression of tTAV, these minigenes limit this phenotype to females. These strains have undergone successful glasshouse cage trials and show potential for application in the field. The diamondback moth and olive fly are the primary insect pests of their respective host crops, brassicas and olives, and cause significant economic damage. Current strategies for their control are primarily reliant on synthetic chemical insecticides, which has led to resistant pest populations, suppression of natural enemies, and the subsequent breakdown of control (Daane and Johnson 2010; Furlong et al. 2013). As such, development of novel, more sustainable, control measures is required.

In transgenic organisms, fitness can be negatively affected by several factors. These include expression of the transgene sequence, insertional mutagenic effects of the transgene insertion, and inbreeding depression, genetic drift or selection related to laboratory adaptation and rearing (Cooley et al. 1988; Bellen et al. 1989; Horn et al. 2002; Uchino et al. 2008; Ahrens and Devlin 2011). In RIDL insects, expression of the transgene sequence comprises the intended expression and off-target expression. For fsRIDL constructs, expression is intended to be lethal to females reared in the absence of the antidote TC (‘off-tet’), while off-target expression might lead to negative effects on males. Similarly, females may be negatively affected by transgene expression even in the presence of TC, if expression of the transgene is not repressed below a harmful level. As several of these effects are specific to the particular transgene insertion, fitness costs may vary between different insertion lines carrying the same transgene but at different chromosomal loci (Lyman et al. 1996; Scolari et al. 2008; Ant et al. 2012; Jin et al. 2013; Yonemura et al. 2013). Previous studies of fitness costs in RIDL/fsRIDL insects have primarily focused on specific behavioral characteristics of homozygous adult males, such as copulation success, induction of female remating refractoriness, longevity, or flight performance, as these are key to the effectiveness of released males (Morrison et al. 2009; Bargielowski et al. 2011a,b, 2012; Ant et al. 2012; Labbé et al. 2012; Jin et al. 2013). Here, however, we chose to assess the cumulative effects of fitness costs over the course of the life cycle and over multiple generations by tracking the evolution of fsRIDL allele frequencies over time. This approach allowed us to estimate whole-life-cycle fitness costs and provided parameter estimates useful in modeling the dynamics of transgene insertion alleles under a variety of scenarios.

We present results from multi-generational laboratory-cage studies measuring the time evolution of fsRIDL alleles in mixed wild-type/transgenic populations of two key pest insect species (diamondback moth and olive fly) reared under either permissive or restrictive conditions. Mass rearing of fsRIDL insects would be conducted under permissive conditions (with TC), whereas released insects and their progeny would face restrictive conditions (no TC). Fitness costs under permissive conditions therefore affect ease and efficiency of rearing and also inform consideration of the likely fate of any hypothetical wild-type allele that somehow entered such a population. Fitness costs under restrictive conditions inform models of the rate of loss of the transgene from a wild population, were releases to cease. Under permissive conditions, no significant fitness costs were found to be associated with OX3097D-Bol, whereas significant selection against the OX4319L-Pxy transgene insertion was evident. Consistent with the predictions of a stochastic simulation model based on the expected female-killing effect of the transgene under such circumstances, both transgenes disappeared rapidly from experimental populations under restrictive conditions.

## Materials and methods

### Experimental conditions and insect rearing

Insects were reared at 25°C, with a 16:8 light/dark cycle. Rearing of diamondback moth and olive fly followed procedures described by Martins et al. (2012) and Ant et al. (2012), respectively. In addition to TC-repressible female-specific lethality, both transgenic strains express a DsRed2 fluorescent protein marker, visible in larvae, pupae, and adults under appropriate filters. OX3097D-Bol was generated using the ‘Demokritos’ strain (Greece) and was later outcrossed for five generations to the ‘Argov’ strain (Israel). Argov was also used as the wild-type strain in this study. OX4319L-Pxy was generated using a diamondback moth colony originating in Vero Beach, FL, USA, which was also used as the wild-type strain in this study. All wild-type strains used have been reared in captivity for >5 years.

The starting frequencies of the transgenic allele in permissive and restrictive rearing experiments were chosen to reflect those expected in extreme examples of two scenarios: (i) wild-type contamination in a mass-reared colony (≥0.5) and (ii) after cessation of inundative releases of fsRIDL insects into the field (0.25). In each experimental generation, the adult insects were housed for 1 week in a 30 × 30 × 30 cm netted cage (Bugdorm, Taichung, Taiwan). Prior to introduction of these insects into the cages, they were sexed and screened as pupae for fluorescence and maintained as separate cohorts until eclosion. When all adults had eclosed, cohorts from each population were placed in their respective cages, with males being introduced first and females 2 h later. Two egg collections were made from each cage during this period, placed on diet and resulting pupae used to found the next generation. As egg collections were taken within approximately the first week after adult eclosion, these experiments did not seek to measure adult fitness costs which manifest after this point. In a mass-rearing setting, adults are rarely kept beyond the first week, when reproductive productivity is highest, and in the wild, mean adult life spans are expected to be <5 days (Furlong et al. 1995).

### Permissive condition experiments

Transgene-permissive conditions were created by providing chlortetracycline (CTC) (for OX4319L-Pxy) and TC (for OX3097D-Bol) in larval diet and adult sugar water (10%) to a final concentration of 100 μg/mL. Olive fly adults were maintained on a yeast–sugar diet without TC. Initial populations with known frequencies of the transgene – OX4319L-Pxy, 0.75; and OX3097D-Bol, 0.5 – were established by crossing transgene-heterozygous males with homozygous females, and transgene-heterozygous males and females, respectively. For diamondback moth, 200 of the resulting progeny from each cage were selected at random as pupae to found the following generation. For olive fly, all pupae surviving in each pot were screened for the DsRed2 marker and the transgenic/wild type ratio calculated for each replicate. The number of transgenics returned to the cage was then made proportional to this ratio, with 200 pupae being selected in total. After egg collections, each cage was frozen and dead adults collected. Of these dead adults, 96 were randomly selected and their gDNA extracted using the following method. Decapitated bodies were placed in individual wells of a 96-well PCR plate, each containing 75 μL of 100 mm NaOH. Plates were heated to 99°C for 30 min in a PCR block, and then, 15 μL of a second solution [250 mm Tris-HCl (pH 8.0) and 0.04% Phenol Red] was added to each well. Samples were genotyped by PCR for presence of the transgene and of the corresponding no-insertion wild-type allele using reactions analogous to those described by Walters et al. (2012) [diamondback moth: 2 min at 94°C, 2 × (10 s at 95°C, 1 min at 62°C, 2 min at 72°C), 26 × (10 s at 95°C, 30 s at 62°C, 30 s at 72°C), and 5 min at 72°C; olive fly: 2 min at 94°C, 35 × (30 s at 94°C, 30 s at 58°C, 50 s at 72°C), and 7 min at 72°C]. One pair of primers was used to amplify sequence spanning the 5′ terminus of the transgene insertion [in diamondback moth ‘OX4319L-Pxy F2′ (sequence available on request) with ‘PB5-out’ (5′-CTCTGGACGTCATCTTCACTTACGTG-3′); and in olive fly ‘OF3097Dforward7′ (5′-CTTACATATAGAGCAGTGCGCTCACATG-3′) with ‘Pb1′ (5′-GGCGACTGAGATGTCCTAAATGCAC-3′)], and another pair was used to amplify sequence spanning the corresponding wild-type locus [in diamondback moth ‘OX4319L-Pxy F2′ with ‘OX4319L-Pxy R1′ (sequence available on request), and in olive fly ‘OF3097Dforward7′ with ‘OF3097D3′reverse4′ (5′-CCTGCGTTTGGAGATGACGAAATC-3′)]. Genotyping results provided estimates of the number of individuals from each of the three genotypes (R/R, R/– and –/–), which was used to calculate generational transgene and wild-type allele frequencies. The experiment was run for 10 generations, with three replicated populations for diamondback moth and two for olive fly.

### Permissive conditions analysis

Under neutral conditions, it is assumed that, having reached Hardy–Weinberg equilibrium, allele and genotype frequencies will remain relatively stable, albeit subject to genetic drift. To test for significant trends in frequency change (a nonstationary process potentially resulting from selection), Mann–Kendall tests were performed. Where these implied that selection was occurring, two further analyses were performed. To test whether significant allele frequency trends could be statistically attributed to selection (rather than drift), a frequency increment test (FIT) was performed on mean generational transgene allele frequencies (Feder et al. 2013). The fitness of individual genotypes was also analyzed by comparing corrected rate of increase (CRI) parameters using a Kruskal–Wallis rank sum test and subsequent *post hoc* testing using Tukey's contrasts. CRI parameters compare observed and expected (under Hardy–Weinberg) genotype frequencies, with the difference between these values indicating the direction and magnitude of selection against this genotype. The mean of these parameters provided an estimated rate of change for each genotype per generation. This allowed for the calculation of mean genotype-specific fitness values (*W*) relative to homozygous wild type. As our best estimates of the fitness costs associate with these transgenes, these values were subsequently used to predict longer-term fsRIDL allele frequency evolution for diamondback moth and olive fly populations. Trajectories were calculated using a recursion model (Hamilton 2009) with initial simulated population genotype frequencies analogous to those used in experimental cages. The model assumes an infinite population size, random mating, constant selection, and no migration. For calculating times to extinction of modeled fsRIDL alleles, a hypothetical population size of 200 individuals was used.

### Restrictive conditions experiments

Transgene restrictive conditions were created by rearing larvae and adults without access to TC sources. For both OX4319L-Pxy and OX3097D-Bol, three replicate populations were analyzed with initial transgene frequencies of 0.25, established by crossing transgene-heterozygous males with wild-type females; this represents the maximum starting allele frequency for a female-lethal transgene in the absence of artificial releases of homozygotes. Replicate populations were observed until the transgene was no longer detected and for one further generation to confirm extinction of the transgene. After pupation, 200 insects from each replicate were randomly selected, then scored for the fluorescent marker and sexed (olive fly as adults). Once eclosed, these adults were placed in new cages to found the next generation. Due to the high level of penetrance of the female-lethal trait in OX4319L-Pxy and OX3097D-Bol under restrictive conditions (>99%, Ant et al. 2012; Jin et al. 2013), and the relatively low initial transgene frequency, it was assumed that all transgenic individuals observed from Generation 2 onwards were heterozygous for the fsRIDL transgene.

### Restrictive conditions analysis

Under restrictive conditions, we hypothesize that the trajectory of fsRIDL transgene frequency change will be most prominently directed by the dominant lethality in females, and the directional selection that this confers. Theoretically, this effect will result in a 50% reduction in fsRIDL allele frequency in each generation relative to the previous. However, this trajectory may be influenced by population-level stochastic effects as well as hypothetical transgene-associated fitness costs. We developed a discrete-generation stochastic model to simulate the potential trajectories of fsRIDL allele decay under situations analogous to our experimental populations (population size of 200, initial fsRIDL allele frequency of 0.25, restrictive conditions). Our model allows for random variation in the sex ratios of the 200 individuals selected each generation. As we assume that only males can carry the fsRIDL allele, this also created variance in the number of individuals inheriting the fsRIDL allele each generation. No fitness costs other than those imparted by female-specific lethality were included, and thus, modeled trajectories represent the potential distribution of trajectories given stochastic variation in population sex ratio and subsequent fsRIDL allele inheritance alone. This was achieved by first estimating the probability that a male in a given generation (*t*) was transgenic *p*(*t*), where the ratio of the number of transgenic males in the previous generation *M*(*t*−1) and the number of total males in the previous generation *N*(*t*−1) is halved, representing the halving of the allele frequency each generation due to female-specific lethality.


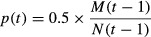
(1)

This probability was then used to calculate the fsRIDL allele frequency in generation *t γ*(*t*). Here *N*(*t*), the number of males in generation *t* was generated using a Binomial (200, 0.5) distribution. The product of *N*(*t*) and *p*(*t*) was then divided by 200 to represent the proportion of transgenic males in the total population and halved to give the allele frequency (as transgenics are assumed to be heterozygotes).


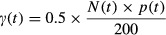
(2)

In Generation 1, fsRIDL allele frequency and sex ratio (male/female) were set at 0.25 and 0.5, respectively, to match the known starting conditions in the experimental populations. Two hundred and fifty independent populations were simulated and allowed to persist until fsRIDL allele extinction. Modeled results were compared to empirical data collected for each species in order to assess whether observed fsRIDL allele frequency decay fell within predicted variation in trajectories, given the assumptions of the model. Subsequently, the mean fsRIDL allele frequency reduction per generation was calculated for both the model and experimental data sets and compared using a Welch two-sample *t*-test. All statistical analysis was performed in R (v. 3.0.02) (R Core Team, 2013). Modeling was performed in Matlab.

## Results

### Selection on the fsRIDL transgene under permissive conditions

**Figure 1:**
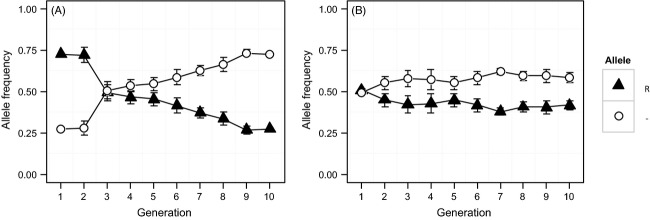
Mean transgene and wild-type allele frequencies (±SE) tracked over 10 generations in three and two mixed populations of diamondback moth (A) and olive fruit fly (B) containing the fsRIDL transgene insertions OX4319L-Pxy and OX3097D-Bol, respectively. Allele frequencies were estimated by genotyping 96 randomly chosen adults per population, per generation, for both the transgene insertion and the corresponding no-insertion wild-type allele. Triangles and circles represent the mean frequencies of the transgene (R) and wild-type (–) allele recorded in each generation, respectively.

**Figure 2:**
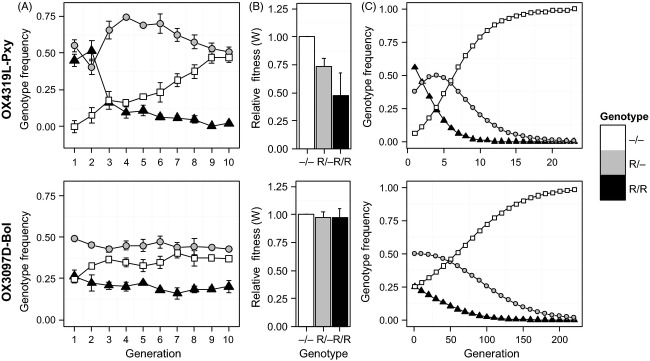
(A) Mean frequencies (±SE) of transgenic homozygous (R/R, triangles), heterozygous (R/–, circles), and wild type (–/–, squares) in two and three mixed-genotype populations of diamondback moth (upper panel) and olive fly (lower panel), respectively. Diamondback moth populations were established with 100 homozygous transgenic male and 100 heterozygous transgenic female insects. Olive fly populations were established with 100 heterozygous transgenic male and 100 heterozygous transgenic female insects. Experimental populations were observed for 10 generations. Engineered female lethality was suppressed throughout the experiment by provision of dietary tetracycline, and population size was maintained at 200 adults in each generation. (B) Relative fitness values for –/–, R/– and R/R genotypes (±SE) of OX4319L-Pxy (upper panel) and OX3097D-Bol (lower panel) calculated from corrected rate of increase parameters with values relative to the –/– genotype. Relative fitness values were *W*_R/R_ = 0.477, *W*_R/–_ = 0.736, and *W*_–/–_ = 1 for diamondback moth and *W*_R/R_ = 0.975, *W*_R/–_ = 0.974, and *W*_–/–_ = 1 for olive fly. (C) Results of a deterministic population genetics model illustrating theoretical genotype trajectories in mixed-genotype populations of diamondback moth (upper panel) and olive fly (lower panel) using the experimentally derived mean estimated relative fitness values from (B). Note that model outputs represent more generations than the cage experiment, to illustrate longer-term trajectories.

In diamondback moth, OX4319L-Pxy transgene allele frequency declined by 63.3%, a trend which was significantly non-neutral (*τ* = −0.956, *P* < 0.01) (Fig. 1A). Frequency increment testing showed a significant departure by the transgene allele frequency from a null, neutral drift distribution (*t*_FITT_ = 2.32, *α* = 0.05). The homozygous wild-type genotype (–/–) showed the highest average increase in frequency, from 0.07 (±0.02 SE) in Generation 2 (the first generation expected to represent genotypes at Hardy–Weinberg equilibrium) to 0.47 (±0.02 SE) in Generation 10 (Fig. 2A). Nonstationary trends were suggested for –/– (tau =0.944, *P* < 0.01) and homozygous transgenic (R/R) (tau = -0.889, *P* < 0.01) genotype trajectories, but not for the heterozygous genotype (R/-) (*τ* = 0.056, *P* > 0.1). Relative fitness values for the R/– and R/R genotypes were calculated to be *W*_R/_– = 0.736 ± 0.07 SE and *W*_R/R_ = 0.477 ± 0.20 SE, respectively (Fig. 2B), with significant differences in CRI calculated between R/R and R/–, –/– genotypes (Stat = −4.968, *P* < 0.01; Stat = −3.509, *P* < 0.01) but not between R/– and –/– genotypes (Stat = −2.068, *P* > 0.1).

In OX3097D-Bol olive flies, transgene allele frequency declined by 15.5%, a significant non-neutral trend (*τ* = −0.664, *P* = 0.012) (Fig. 1B). However, no significant departure from a neutral drift distribution was observed for mean transgene allele frequencies at *α* = 0.05, although a significant difference was observed at *α* = 0.15 (*t*_FITT_ = 1.018). Partitioning this behavior to the genotype level, the –/– genotype showed the greatest mean increase, rising from an initial frequency of 0.25 to an average of 0.37 (±0.05 SE) after 10 generations (Fig. 2A), a trend which was significantly non-neutral (*τ* = 0.584, *P* = 0.024). However, trajectories of R/– and R/R genotype frequencies showed no significant trend (*τ* = −0.477, *P* = 0.071; *τ* = −0.576, *P* = 0.057). Relative fitness values of *W*_R/R_ = 0.974 ± 0.07 SE and *W*_R/–_ = 0.975 ± 0.05 SE were calculated (Fig. 2B); however, genotype was not found to significantly explain differences in CRI values (KW^*χ*2^ = 0.730, *P* > 0.1).

Relative fitness parameters estimated from these empirical data were used to predict changes in genotype frequency over time (Fig. 2C). In all projected genotype trajectories, selection led to the eventual fixation of the wild-type allele and the concomitant loss of the transgene. For OX4319L-Pxy, comparatively high transgene-associated fitness costs led to a predicted wild-type allele fixation – where the modeled number of transgenic individuals falls to <1 – within 30 generations (initial wild-type allele frequency = 0.25), while the estimation of much lower transgene-associated fitness costs for OX3097D-Bol resulted in predicted wild-type allele fixation after approximately 200 generations (initial wild-type allele frequency = 0.5).

### Selection on the fsRIDL transgene under restrictive conditions

**Figure 3:**
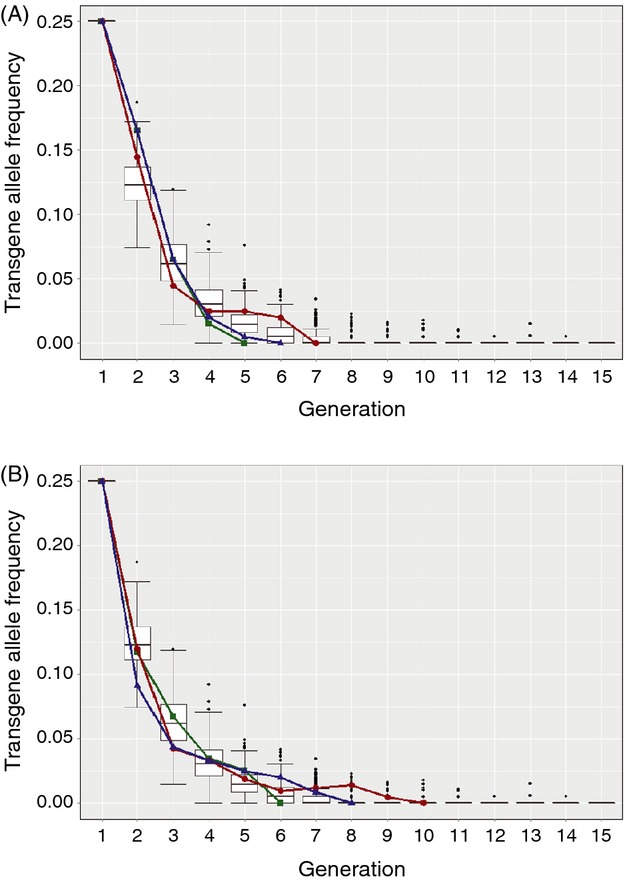
Boxplots showing results from 250 iterations of a stochastic model simulating engineered female-specific selection on a fsRIDL allele in a panmictic, closed population of constant size over 15 discrete generations. We consider fsRIDL allele frequency with a starting population of 200 individuals and an initial fsRIDL allele frequency of 0.25 (*f* = 0.25) propagating in the absence of the transgene repressor (under restrictive conditions). Horizontal bold lines represent generational medians; upper and lower box lines represent first and third quartiles, respectively; outer horizontal lines represent 1.5× the interquartile range; and dots represent data points over 1.5× above or below the first and third quartiles, respectively. Overlaid onto the boxplots are lines (red, blue and green) showing allele frequency changes from three replicates of caged experiments tracking fsRIDL allele frequencies in mixed populations of (A) diamondback moth and (B) olive fly reared under analogous conditions to those used in the model (initial fsRIDL allele frequency of 0.25, restrictive conditions).

Given an initial transgene allele frequency of 0.25 in a closed population of 200 individuals under restrictive conditions, our stochastic model predicted fsRIDL transgene extinction within nine generations in approximately 95% of iterations, with a mean and maximum number of generations until allele loss of 6.5 (±1.8 SD) and 15, respectively (Fig. 3). fsRIDL allele frequency decay in experimental populations fell well within the variation predicted by this stochastic model. Mean number of generations until disappearance of the fsRIDL allele in diamondback moth and olive fly populations was 6.0 (±0.58 SE) and 8.0 (±1.16 SE), respectively, with respective maximum number of generations until allele extinction of 7 and 11. On average, OX4319L-Pxy allele frequency decreased in each generation by 50% (±6.0 SE), while OX3097D-Bol frequency decreased by 45% (±4.9 SE). Modeled mean fsRIDL allele frequency fell by 47% (±7.2 SE) per generation and did not significantly differ from either experimental estimates (OX4319L-Pxy: *t* = −0.260, *P* > 0.1; OX3097D-Bol: *t* = 0.211, *P* > 0.1).

## Discussion

Under permissive conditions, we measured significant negative transgene allele frequency trends over the experimental period in both fsRIDL constructs in their respective host strains (Fig. 1). In diamondback moth populations, these frequency changes could be attributed to selection against the transgene. However, in olive fly, transgene allele frequency changes could not be significantly differentiated from neutral drift (at *α* = 0.05, although differences at *α* = 0.15 showed significance). As our experimental population size (*N*) was substantially larger (>20×) than the number of generations observed, however, we assume that genetic drift (which changes allele frequencies over periods of approximately *N* generations) is unlikely to have significantly affected our results in this experiment (Illingworth et al. 2012). If transgene-associated fitness costs are very low (as may be the case in OX3907D-Bol), increased replication (number of observed generations) relative to that used here may therefore be required to differentiate selection from neutral drift.

In OX4319L-Pxy, negative transgene allele frequency trends were primarily driven by selection against R/R individuals. This was the only genotype to show both significant negative frequency trends and CRI values significantly different from –/– (Fig. 2A,B). In OX309D-Bol, neither transgenic genotype showed a significant frequency trend (although interestingly a significant positive trend was observed for the –/– genotype) nor was genotype found to significantly explain variation in CRI values (Fig. 2A,B). If very low fitness costs are associated with this strain, further replication may be required to elucidate their presence. These findings are consistent with those from previous studies of these strains exploring different measurements of relative mating competitiveness when in competition with nontransgenic counterparts. For OX4319L-Pxy, previous laboratory-cage assays of mating initiation and progeny share (thus including postcopulatory selection) indicated small fitness costs associated with transgene-homozygous males relative to males of their wild-type background strain (Jin et al. 2013). On the other hand, glasshouse-based studies using homozygous OX3097D-Bol males and wild-type olive flies suggested the absence of transgene-related fitness costs to mating behavior. These results were further supported in experiments showing strong mating and remating competitiveness of OX3097D-Bol males when compared with field-collected wild insects and the successful suppression of caged wild-type olive fly populations in a simulated release scenario (Ant et al. 2012).

As this experiment took into account a wider range of factors than previous studies on these lines (in terms of behaviors and sexes analyzed), a lower estimate of overall fitness might have been anticipated. In particular, fitness costs for a female-lethal strain, even under permissive conditions, might be significantly higher in females than in males. In fact, the estimates of relative R/R fitness in this study (OX3097D-Bol = 0.975, OX4319L-Pxy = 0.477) (Fig. 2B) did not differ greatly from estimates of relative mating competitiveness and progeny share calculated previously for homozygous males from these strains [OX3097D-Bol = 1.083 (T. Ant, M. Koukido and L. Alphey, unpublished manuscript), OX4319L-Pxy = 0.580] (Ant et al. 2012; Jin et al. 2013). As these estimates of relative fitness are well above minimum recommendations for an efficient autocidal release program (FAO/IAEA/USDA 2003), these results reinforce the potential of these strains to be employed in such a control strategy.

Beyond their potential impacts on suppression efficacy, the transgene-associated fitness costs present in these two fsRIDL strains have a number of implications at the population level. Using our best estimates of the fitness costs associated with these lines (derived from this study), our population modeling suggests that even under conditions where engineered lethality is suppressed, competition between transgenic and wild-type alleles will result in gradual increase in frequency of the wild-type (noninsertion) allele (Fig. 2C). However, the relatively low fitness cost of the transgene, especially for OX3097D, suggests that a wild-type allele somehow entering a mass-rearing colony would spread only slowly. This is a significant improvement over classical translocation-based sexing strains, for example the medfly *tsl* strains, which have severe fitness costs due to aneuploidy in offspring (Fisher 2000). We assume that the primary cause of these transgene-associated fitness costs, where present, is insertional mutagenesis and/or incomplete female specificity of the fsRIDL phenotype. The contributions of other components of the constructs, however, such as the DsRed2 fluorescent protein marker, cannot be ruled out. However, the differing rates of selection against the two constructs, both of which include the DsRed2 gene under the control of the same promoter/enhancer, indicate that detected fitness costs are likely not primarily due to the marker.

Although no significant fitness costs associated with R/– were evident in either strain under permissive conditions, we expected that fitness costs would be more marked under restrictive conditions. We compared fsRIDL allele evolution between modeled populations in which the sole selection force on fsRIDL insects was engineered female-lethality, and empirical data collected under analogous conditions. In both species, experimental fsRIDL allele decay fell well within that predicted by our stochastic model (Fig. 3). Average generational fsRIDL allele frequency reductions in both lines did not differ significantly from those predicted by our stochastic model. These results indicate that fitness costs to heterozygote males (as carriers of fsRIDL alleles under restrictive conditions), if present, have little effect on allele frequency evolution under these conditions. As predicted, both transgenes went extinct in all experimental populations within a small number of generations, indicating that transgene persistence in the field would be relatively transient in the absence of ongoing releases of additional transgenics, even from a high starting allele frequency. Even if some individuals had access to permissive conditions, that is, high levels of TC, during larval development – implausible in the field for these insects (Kumar et al. 2005; Hu et al. 2010; Seo et al. 2010) – most would likely not, and the fitness cost of the transgene under both restrictive and permissive conditions would ensure rapid disappearance of the transgene from the population. It is likely that the dynamics of these fsRIDL transgenes in a postrelease field population will be influenced to some degree by aspects of the local genetic background. In general, the local genetic background is expected to be better adapted to local conditions than the genetic background of a strain that has been reared in captivity for many generations. Linkage with maladaptive background alleles might lead to an initial reduction in transgene frequency that is slightly more rapid than seen in our experiments using similar backgrounds for wild-type and transgenic strains. This could be addressed by comparison against wild-caught or more recently colonized strains in realistic environmental settings, perhaps in small-scale release experiments.

Our data show that protein-coding transgenes can impose a low fitness cost. Fitness costs due to transgene expression would be expected to be dominant or codominant, so it is particularly striking that the observed fitness costs were substantially recessive. This is somewhat surprising for a conditional lethal transgene and suggests that basal expression of the lethal effector is relatively harmless. In contrast, taking a similar multigenerational approach, Paton et al. (2013) identified strong selection against a synthetic malaria-refractory transgene (EVida3) in the mosquito *Anopheles gambiae*, resulting in extinction of the allele in all replicate populations by Generation 10. Significant fitness costs to the larval stages of these transgenic mosquito strains were identified, possibly due to unintended background transgene expression. The much longer predicted persistence times of our lethal transgenes under permissive conditions suggests that it is possible to design constructs and generate transgene insertion sites where such background fitness costs can be minimized. This is encouraging both for the rearing and use of these strains, and also for the development of ‘self-sustaining’ strains and strategies in which, unlike fsRIDL, the transgene is intended to persist in the environment for many generations or indefinitely after releases cease. In particular, it suggests that it may be possible to deploy such transgenes effectively, at least on trial scales, by inundative release. Even though the transgenes would eventually disappear (due to associated fitness costs), and so the approach is self-limiting, the transgene may persist in closed populations at a useful frequency for many generations (Gould et al. 2008; Rasgon 2009). This would allow effector genes and molecules to be tested in the field without requiring coupling to gene drive systems, which are more challenging from both technical and regulatory perspectives. The findings of this study support the further development, testing and use of genetic control methods and, in particular, of the fsRIDL strains and systems examined.

## Data archiving statement

Data for this study are available from the Dryad Digital Repository: http://dx.doi.org/10.5061/dryad.40gg7.
